# Smoking Cessation with Medication and Behaviour Therapy

**DOI:** 10.12669/pjms.336.13653

**Published:** 2017

**Authors:** Memet Taskin Egici, Guzin Zeren Ozturk, Mulazim Hussain Bukhari, Dilek Toprak

**Affiliations:** 1 Memet Taskin Egici, MD. Family Medicine Clinic, Sisli Hamidiye Etfal Training and Research Hospital, Istanbul, Turkey; 2 Guzin Zeren Ozturk, MD. Family Physicians, Sisli Hamidiye Etfal Training and Research Hospital, Istanbul, Turkey; 3 Mulazim Hussain Bukhari Head of Pathology Department, University of Lahore, Pakistan; 4 Dilek Toprak, MD, Associate Professor, Department of Family Medicine, Family Medicine Clinic, Sisli Hamidiye Etfal Training and Research Hospital, Istanbul, Turkey

**Keywords:** Smoking Cessation, Smoking, Tobacco, Smoking Cessation Medicine, Smoking Cessation Clinic, Behavioral Therapy

## Abstract

**Background and Objective:**

Smoking Cessation Clinics (SCCs) involve the use of cognitive behavior therapy and effective medications provided by specialists. Our objective was to report socio-demographic data, investigative services provided, and determine the smoking cessation success.

**Methods:**

Data from all hospitals affiliated with the Beyoglu Public Hospitals Union were obtained between January 1, 2015 and December 31, 2015. Data from Sisli Hamidiye Etfal Training and Research Hospital were reviewed via retrospective chart review in the same period. Frequency and average values were determined using statistical software. In the evaluation of related factors, chi-square and student t tests were applied; p ≤ 0.05 was considered statistically significant.

**Results:**

The mean admission age was 38.72 ± 12.20 years (min 13; max 94). Women tended to be older than men at the time of admission but men started smoking younger than women. Less than the high school educated subjects started smoking at early ages. The smoking cessation rate was 39.3% (n = 219) when treated with medication and behavioral therapy.

**Conclusion:**

About 48% smokers stopped smoking after treatment with medications and behaviour therapy. Most of the smokers were between 30-50 years of age. More Smoking Cessation Clinics should be established to allow access to more highly educated patients to smoking cessation resources.

## INTRODUCTION

Smoking addiction is a serious public health problem worldwide. The World Health Organization (WHO) defines smoking as “the world’s fastest-spreading and longest-running epidemic”.^1^ The United States Department of Health’s report states that smoking is addictive, nicotine is an addictive substance in cigarettes, and nicotine dependence is similar to heroin and cocaine dependence.^2^ There are currently 1.1 billion smokers over the age of 15 worldwide.^3^

The first legal regulation in the fight against tobacco in Turkey was made in 1996. In 2004, the WHO Framework Convention on Tobacco Control was put into practice. In 2007, the Provincial Tobacco Control Boards were established in 81 provinces so that the implementation of the National Tobacco Control Program as a whole, including follow-up activities, could be implemented in the provinces.^4^ In 2008, 5727 amending Law No. 4207, the “Law Amending the Law on Prevention of Harmful Effects of Tobacco Products” was adopted. On July 19, 2009, all enclosed areas in Turkey were made smoke-free by including all indoor areas (e.g., restaurants, cafes, and bars).

Epidemiological data show that 70% of smokers want to quit smoking, and 46% have attempted to quit at least once.^5^ It is important to support those who want to quit smoking both psychologically and medically.^6^

In Turkey services are provided for nicotine dependence reduction as well as for smoking cessation. Components of the system that help smokers quit include brief referrals and withdrawal assistance lines as well as smoking cessation clinics involving the use of cognitive behavior therapy and effective medications provided by experienced specialists.^7^

The smoking cessation clinic (SCC) at our hospital was previously run by both psychiatry and family medicine departments until 2015 but has subsequently shifted to the family medicine clinic. Our objective was to report socio-demographic data, investigate services provided, and determine the smoking cessation success of our SCC at the Sisli Hamidiye Etfal Training and Research Hospital.

## METHODS

This retrospective descriptive study was performed on 4.119 smokers admitted between January 2015 and December 2015 to SCCs of hospitals of Beyoglu Public Hospitals Union (BPHU) in Istanbul. There was no need for ethical approval because the study was retrospective so that’s why permission was taken from BPHU General Secretariat only.

Hospitals which are member of BPHU and have common electronic database in addition to SCCs were included in this study: Sisli Hamidiye Etfal Training and Research Hospital, Okmeydani Training and Research Hospital, Gazi Osman Pasa Taksim Training and Research Hospital; Balta Harbor Bone Diseases Training and Research Hospital, and Eyup State Hospital. Required data were collected from SCCs and BPHU General Secretariat database. Sisli Hamidiye Etfal Training and Research Hospital (SHETRH) SCC data were reviewed via retrospective chart review in the same period because it was our main SCC. All records in that period were included in the study Frequency and average values were determined using statistical software. In the evaluation of related factors, chi-square and student t tests were applied; p ≤ 0.05 was considered statistically significant.

## RESULTS

In 2015, a total of 4.119 patients were referred to the SCC affiliated with the General Secretariat of BPHU. There were 2.443 (59.3%) male patients and 1.676 (40.7%) female patients. The minimum age was 13, the maximum age was 95, and the mean age was 39.73 ± 12.39 years. The mean age of female patients was 41.22 ± 11.76 years, and the mean age of male patients was 38.71 ± 12.72 years.

The most frequent admission was in the age group of 35–49 (n = 1569, 38.1%). Males were most frequently in the 20–34 year age group, while females were most frequently in the 35-rs and 26 of these (53.06%) were female.

The mean number of admissions per patient to SCCs was 1.55 ± 0.98 times/year. When the responsible physicians of SBPs were evaluated according to specialty, the distribution of patients were 2355 (57.2%) family physicians, 1424 (34.6%) pulmonologists, and 340 (8.3%) psychiatrists.Table-I.

**Table-I:** Distribution of the doctors responsible for Smoking outpatient clinics according to specialty

	*Numbers (Percentage)*
Family Medicine	2355 (57%)
Chest Diseases	1424 (35%)
Psychiatry	340 (8%)

When the distribution of applicants to these centers was examined, most admissions were made to Sisli Hamidiye Etfal Training and Research Hospital (SHETRH; 41.3%, n = 1700). A significant number of patients (1360, 80%) were admitted to family medicine’s SCC. One thousand-forty-five (61.5%) of these patients were male, and 655 (38.5%) were female. The minimum admission age was 13 years, the maximum was 94 years, and the mean age was 38.72 ± 12.20 years. Twenty-one (21.2%) patients under the age of 18 applied. The average age of males was 37.70 ± 12.42 years, while the average age of females was 40.34 ± 11.66 years. The most frequent admission was in the 20–34 age group (41.1%, 69.8%). Table-II

**Table-II:** Distribution of patients according to hospitals.

	*Numbers (Percentage)*
Sisli Hamidiye Etfal Training and Research Hospital	1700 (42%)
Okemydani Training and Research Hospital	1300 (32%)
Gazi Osman Pasa Taksim Training and Research Hospital	351 (9%)
Balta Lamani Bone Diseases Training and Research Hospital	439 (11%)
Eyup State Hospital	231 (6%)

According to family medicine-related SCC data from SHETRH, 524 applicants were female (38.7%) and 834 were male (61.3%). The mean age was 39.35 ± 12.17 years (minimum 15, maximum 83 years). The mean age of females was 41.18 ± 11.44 years (minimum 16, maximum 83 years), while it was 38.20 ± 12.47 years (minimum 15, maximum 77 years) in males. According to age group, most smokers were in the age group of 35–49 years. Females were more likely to be in the 35–49 year age group (249, 47.3%) and males in the 20–34 year age group (344, 41.2%). There was a significant relationship between gender and age (p = 0.000). Women tended to be older than men at the time of admission.

While 788 (51.2%) smokers admitted to SHETRH were high school educated or above, 582 (42.8%) received less education than high school. Sixteen (1.2%) patients were illiterate. The education level of males was found to be significantly higher than females (p = 0.000). We found that the level of education decreased significantly with increasing age at admission (p = 0.000).

While 810 (59.1%) of those who came to the SCC were single, at least one person smoked in the family were 98.8% (n = 1.344). There was no significant relationship between marital status and number of smokers in the family (p = 0.783).

When the age of smoking initiation was questioned, the mean age was 17.40±5.20 years (minimum 7, maximum 60 years). According to gender, smoking initiation began in females at 18.38 ± 5.75 years, while in males it was found to be 16.79 ± 4.72 years, and this was statistically significant (p = 0.000). When the educational status (high school and above, high school) and the age at which the person started to smoke were compared, it was found that the age at which smoking began was higher in the high school and above educationed subjects (p = 0.000).

The mean pack/year among applicants to SCCs was 21.99 ± 13.70 (minimum 1, maximum 80 pack/year). When the relationship between gender and number of cigarettes (pack/year) was examined, the pack/year average of females was significantly higher than the pack/year average of males (p = 0.046). When the relationship between educational status (high school and above, high school) and the number of cigarettes smoked (pack/year) was examined, the number of cigarettes smoked (pack/year) was lower in high school and above education (p = 0.000).

About 44.9% (n = 1423) of the patients who had medication and cognitive behavioral treatment were quit smoking when all hospitals belonging to the BPHU General Secretariat were taken into consideration, and patients who were not discharged were not evaluated. The smoking cessation rate was 39.3% (n = 219) when treated with medication and behavioral therapy.

## DISCUSSION

The frequency of tobacco product use among individuals 15 years and older in Turkey is 27.1%. This value corresponds to 14.8 million people. Monso et al found Male gender and older age to be effective in smoking cessation.^8^ According to the Turkish Statistical Institute, the use of tobacco products is higher in males (41.5%) than in females (13.1%). The majority of users of tobacco products (94.8%) were smoking cigarettes and only 0.8% used hookahs.^9^ In our study, the majority of patients (59.3%) were male when all hospitals were evaluated. Yasar et al. found that 60.2% of patients who visited the cigarette polyclinic were found to be male in their study.^10^ The number of patients admitted in another study was 127; 30.7% were female and 69.3% were male.^11^ SHETRH’s SCCs patients were primarily male (61.5%). When SCC admission data (according to family medicine) were examined, males were more likely to be admitted (61.3%). As the rate of smoking is higher in males than in females in our country (mirroring global trends), it is expected that the number of male patients being admitted is higher than females.

SCC admissions are most frequently patients in the 25–44 year age group according to a study from USA.^12^ In our study, the average age of application to all SCCs was 39.73 ± 12.39, and the average age group was found to be 35–49 years old. When we look at smoking cessation outpatient clinic related to SHEEAH Family Physician, most patients were in the age group of 35–49 years. This may be due to the start of the harmful effects of cigarettes, the frequent occurrence of additional illnesses, and the possibility of marriage in this age group.

Females who applied to the SHEEAH’s SCC were found to be significantly older than males (p = 0.000). At the same time, the age at which males started to smoke was less than that of females (p = 0.000). Similarly, in a study published in 2012, the average age of onset of cigarette smoking was 17 (6–37) years, and the mean age of onset of smoking in females was 18.09 ± 5.14 years and 16.09 ± 4.43 years in males.^13^ Based on these data, we conclude that considering the age at which a patient applies for cessation resources is important as the age at which females start smoking is higher than males.

According to one study, 58.7% of cigarette smokers are smoking before 18 years of age, the legal age at which one can purchase cigarettes.^12^ When all hospitals in the region were taken into consideration, the number of smokers under the age of 18 was 49 (1.2%), and 26 (53.06%) were females. We believe that young people begin smoking because they perceive smoking to be an expression of growth and freedom and because of a desire to be involved in a group.

According to 2010 data, smoking is lower in females with lower educational levels, and smoking rates also increased as education level increased.^14^ In a study published in 2017, smoking rates were found to increase with lower educational attainment.^15^ In our study we found a relationship between educational status and the number of cigarettes per person smoked annually. We believe this is due to the increased level of education regarding the harmful effects of smoking.

Smoking cessation treatment consists of behavioral therapy and motivation as well as pharmacological treatment.^12^ Studies have shown that both are effective alone, but the success rate increases when used together.^16^

According to statistical data of our country, 13.6% of those who attempted to quit smoking used pharmacologic treatment. 8% of those who attempted to quit were given counseling, and most (73.4%) attempted to quit without any help. 9

The smoking cessation rate was 39.3% (n = 219) when treated with medication and behavioral therapy. In Turkey, smoking cessation drugs were provided by the SCC free of charge to the patient. Free drug treatment may increase individual motivation to quit smoking.

## CONCLUSION

Smoking cessation treatment is a special treatment consists of cognitive behavioral therapy and motivation as well as pharmacological treatment. Smoking Cessation Clinics are the most important centers for smoking cessation and prevention from cigarette related diseases. The widespread use of SCCs is important both in terms of individual and social wellbeing. For this reason, necessary certification trainings should be organized, and new SCCs should be opened to allow access of more highly educated patients to smoking cessation resources.

### Authors’ Contributions

***MTE*** conducted this research and is responsible and accountable for the accuracy and integrity of this work.

***GZO*** helped in collection of data and its interpretation.

***MHB*** reviewed the manuscript.

***DT*** helped in data analysis.
